# Special Issue “Molecular Basis and Treatment of Skin Diseases and Their Associated Complications”

**DOI:** 10.3390/ijms27052326

**Published:** 2026-03-02

**Authors:** Vincenzo Piccolo, Maria Maisto

**Affiliations:** NutraPharmaLab, Department of Pharmacy, University of Naples “Federico II”, 80131 Naples, Italy; vincenzo.piccolo3@unina.it

For decades, chronic skin diseases were considered conditions limited to the epidermis. However, emerging scientific evidence reveals a more complex and interconnected pathological framework [1,2,3]. Cutaneous inflammation is closely linked to several systemic alterations, including genetic and epigenetic predispositions, immunometabolic alterations, the gut–skin axis communication, and the intracellular molecular pathways that regulate growth and repair [4,5,6,7,8]. The papers collected in this Special Issue exemplify this evolving perspective, demonstrating how dermatological research is increasingly bridging molecular biology, immunology, and systemic medicine. In this scenario, to refine the management of chronic inflammatory skin diseases, Hernandez-Bello et al. used a case–control design to explore genetic contributors to immune dysregulation in plaque psoriasis (Contribution 1). Specifically, the authors examined how two different *FOXP3* gene variants (rs2280883 and rs3761548) play a pivotal role in T-cell regulation through their effects on anti-inflammatory cytokines interleukin-10 (IL-10) and transforming growth factor-β1 (TGF-β1) production levels and their association with plaque psoriatic risk. The authors, by comparing 101 patients with psoriasis with 106 healthy controls, found that the *FOXP3* rs2280883 AA genotype conferred a significantly increased risk of disease, whereas rs3761548 showed no association. The IL-10 levels were elevated in patients, but neither IL-10 nor TGF-β1 correlated with *FOXP3* polymorphisms, suggesting that these variants influence disease primarily through localized immunoregulatory mechanisms rather than systemic cytokine modulation. Continuing within the context of psoriasis research, Di Caprio et al. explored the bidirectional crosstalk between psoriatic inflammation and adipose tissue (Contribution 2). Using an ex vivo human model, the authors stimulated subcutaneous adipose tissue fragments with interleukin-17 (IL-17) and tumor necrosis factor-α (TNF-α), two hallmark cytokines of the psoriatic immune axis, and examined the related effects on adipokines and inflammatory mediators. The study showed that psoriasis-related cytokines drive adipose tissue toward a pro-inflammatory phenotype by reducing adiponectin (an anti-inflammatory adipokine) and increasing resistin and multiple inflammatory mediators (IL-6, IL-8, IL-23, and IL-36γ). In line with this systemic perspective, Radhakrishna et al. investigated the biological basis of chronic pain in hidradenitis suppurativa (HS), a recurrent inflammatory skin disorder characterized by painful nodules and abscesses in apocrine gland areas (Contribution 3). Using a genome-wide DNA methylation approach, an epigenetic mechanism that regulates gene activity without altering the DNA sequence, the authors analyzed DNA extracted from peripheral blood leukocytes, thereby capturing systemic epigenetic signatures associated with chronic pain. They identified 253 differentially methylated CpG sites (cytosine–phosphate–guanine regions regulating transcription), predominantly hypomethylated, across genes involved in nociception, neurotransmission, oxidative responses, and immune regulation. Notably, altered methylation in ion channel genes such as *CACNA1C* (calcium signaling) and *SCN9A* (sodium channel linked to pain perception) indicated enhanced neuronal excitability. Thus, the authors concluded that pain in HS reflects a lasting biological reprogramming rather than a simple symptomatic event, highlighting the need for targeted therapeutic strategies beyond conventional anti-inflammatory care. Continuing this systemic perspective on cutaneous inflammation, Jang et al. examined how chronic gut inflammation and microbial dysbiosis contribute to atopic dermatitis (AD) through the gut–skin axis (Contribution 4). The authors used a TNBS-induced irritable bowel syndrome (IBS) mouse model to reproduce chronic colonic inflammation and gut barrier dysfunction. They subsequently treated the mice with topical MC903 (calcipotriol, a vitamin D3 analogue) to induce AD-like skin lesions. The IBS mice exhibited marked colon inflammation, disrupted tight-junction proteins, and increased intestinal permeability, together with elevated circulating interleukin-6 (IL-6) and lipopolysaccharide (LPS) levels, indicating systemic inflammation. Their results indicated that IBS mice developed more severe AD-like dermatitis after challenge with MC903, with increased scratching behavior, greater epidermal thickening, and higher TSLP and IL-6 receptor expression in the skin compared to the control group. Altogether, the authors concluded that chronic intestinal inflammation and microbial imbalance can potentiate AD by weakening the gut barrier, promoting systemic inflammation, and sensitizing the cutaneous immune response, highlighting the intestine–skin axis as a promising therapeutic target in atopic dermatitis. Other evidence emerged from the work of Papaccio et al., who investigated whether vitiligo, a condition historically regarded as limited to melanocyte loss, also presents systemic metabolic and inflammatory alterations (Contribution 5). Using a case–control design with 50 patients with vitiligo and matched healthy controls, the authors assessed serum and plasma lipid profiles, micronutrients, oxidative stress markers, cytokines, and fatty-acid composition. The patients with vitiligo showed increased LDL cholesterol and reduced vitamin D and folate levels, along with altered homocysteine metabolism and decreased superoxide dismutase activity, accompanied by elevated advanced glycation end-products, indicating persistent oxidative stress. Higher circulating IL-6 and CXCL10 levels further confirmed systemic inflammation, while lipidomic analysis revealed a shift toward pro-inflammatory n-6 polyunsaturated fatty acids and a relative reduction in n-3 polyunsaturated fatty acids. Taken together, these findings suggest that vitiligo is associated with measurable systemic metabolic and inflammatory dysregulation, supporting the importance of considering cardiometabolic risk and adopting a more integrated clinical management approach in affected patients. In addition to immunometabolic and neuroimmune dysregulation, this Special Issue expands the concept of skin disease as a locus of biomechanical and vascular pathology. Yuan et al. obtained compelling experimental evidence that mechanical-stress-driven skin injury is not a limited local ischemic event but rather a thrombo-inflammatory process that needs to be targeted for intervention. In a rat model of pressure injury, the authors demonstrated that topical prophylaxis with the low-molecular-weight antiplatelet agent trapidil significantly reduced ulcer incidence and severity by attenuating post-decompression thrombosis and microvascular dysfunction, without altering systemic coagulation. These findings emphasize that cutaneous damage arising from mechanical load shared pathogenic themes with chronic inflammatory dermatoses, namely, endothelial activation, impaired perfusion, and sterile inflammation, opening avenues for local vascular-protective strategies in dermatologic surgery and wound prevention (Contribution 6). Jeong and Lee explored a previously understudied regulatory layer in atopic dermatitis involving dysregulation of the Hippo pathway (a mechanotransduction and growth-control signaling cascade) and its effector YAP (Yes-associated protein) (Contribution 7). The authors found that AD skin exhibited nuclear YAP accumulation, which amplified inflammatory signaling and keratinocyte proliferation. Pharmacologic inhibition of YAP with verteporfin suppressed Th2- and Th1-mediated cytokine expression (including IL-4, IL-6, IL-13, and IFN-γ), reduced mast-cell infiltration, and normalized epidermal hyperplasia. Mechanistically, YAP activation was linked to enhanced JAK–STAT signaling (Janus kinase/signal transducer and activator of transcription), a key axis regulating cytokine-driven immune responses in AD, indicating that transduction-immune crosstalk plays a central role in disease amplification. Within this Special Issue, Parab et al. provide further insight into the systemic nature of cutaneous malignancy, examining melanoma progression in a syngeneic murine model carrying the *BRAF^V600E^* mutation, a constitutive activating variant of the *BRAF* oncogene that persistently drives the MAPK/ERK (mitogen-activated protein kinase/extracellular-signal-regulated kinase) pathway and catalyzes uncontrolled melanoma cell proliferation (Contribution 8). The authors profiled the mediators involved in melanoma progression during the early and advanced stages using single-nuclei RNA sequencing to avoid dissociation-induced alterations. They found that increasing intratumoral heterogeneity, with melanoma cells progressively adopting invasive, neural-crest-like, and hypoxia-associated programs. In parallel, the tumor microenvironment shifted toward immunosuppression and angiogenesis, evidenced by the expansion of the proportion of macrophage subsets expressing VEGFA (vascular endothelial growth factor A) and a decline in antigen-presenting populations. Overall, the study highlights melanoma progression as a coordinated process involving oncogenic signaling, metabolic stress, and myeloid-driven immune remodeling, supporting therapeutic approaches that co-target tumor cells and their supportive microenvironment.

In addition to such original research contributions, this Special Issue encompasses mechanistic review articles that further consolidate the knowledge that chronic inflammatory skin diseases represent complex systemic disorders rather than exclusively cutaneous entities. Collectively, these reviews elucidate converging biological mechanisms, including cytokine-mediated crosstalk, immunometabolic reprogramming, neuroimmune activation, and multi-organ communication, which ultimately shape disease onset, progression, and systemic comorbidity profiles. Artusa et al. conducted a comprehensive overview of pediatric atopic dermatitis (AD), reporting that the disorder extends beyond primary epidermal barrier impairment to encompass interconnected immune, neurogenic, and psychosocial alterations (Contribution 9). The authors highlight how genetically driven barrier dysfunction and cutaneous–gut microbiota imbalance induce an aberrant activation of Th2 (T-helper) and Th17 pathways. Th2 cytokines (IL-4, IL-5, IL-13) drive IgE class switching, eosinophilic recruitment, and further barrier disruption, while Th17 mediators (IL-17, IL-22) promote keratinocyte activation and neutrophilic inflammation. Particular emphasis is placed on IL-31 as a key pruritogenic cytokine mediating neuronal hypersensitivity and itch transmission. Pro-inflammatory cytokines, including IL-1, IL-6, and TNF-α, further maintain the chronic inflammatory condition. Finally, the review underlines the links between this immunological alteration and sleep disturbance, cognitive impairment, and behavioral dysregulation, framing pediatric AD as a multifaceted immune-neuro-psychodermatological condition warranting multidisciplinary management. Extending the systemic framework, Joo et al. examined how dysregulated PI3K/Akt/mTOR (phosphoinositide-3-kinase/protein kinase B/mechanistic target of rapamycin) signaling underpins both the cutaneous and extra-cutaneous manifestations of psoriasis (Contribution 10). Their analysis highlights mTOR as a critical metabolic hub integrating nutrient sensing and inflammatory signaling to drive keratinocyte hyperproliferation, synovial activation in psoriatic arthritis, and endothelial dysfunction associated with cardiovascular comorbidity. This molecular axis operates synergistically with Th17-related cytokines (IL-17 and IL-22) and TNF-α, creating a self-reinforcing inflammatory–metabolic loop. The authors proposed mTOR modulation as a promising therapeutic avenue capable of addressing the cutaneous burden alongside systemic metabolic and vascular complications. In a complementary direction, Papa et al. examined the emerging “skin–bone axis,” emphasizing how chronic dermatologic inflammation impairs skeletal integrity. The review details how IL-17, IL-1β, and TNF-α synergistically induce RANKL (receptor activator of NF-κB ligand)-dependent osteoclastogenesis and suppress Wnt (wingless-related integration site)/β-catenin (beta-catenin) pathway activity, thereby promoting bone resorption while limiting osteoblast activity (Contribution 11). Vitamin D deficiency, gut microbiota alterations, and microRNA-mediated epigenetic regulation were identified as modulators exacerbating immune–skeletal dysregulation. By integrating immunological, endocrine, and microbial perspectives, the authors position inflammatory skin disorders as systemic conditions that predispose patients to osteometabolic fragility and identify vitamin D supplementation, microbiome-targeted interventions, and miRNA therapeutics as emerging strategies. Further reinforcing the systemic paradigm, Hołdrowicz and Żebrowska delineated the molecular interface between psoriasis and depression, focusing on the bidirectional skin–brain axis (Contribution 12). Their synthesis demonstrates how persistent cutaneous inflammation instigates neuroinflammatory cascades via IL-6-mediated hypothalamic–pituitary–adrenal axis activation, IL-17-driven microglial sensitization, and TNF-α-induced compromise of blood–brain barrier integrity. Additionally, shared immunogenetic alterations, including altered B-cell signaling via CD19 and overlapping cytokine modules, support a mechanistic link beyond psychosocial burden. This framework highlights the need for early psychological assessment and integrative psycho-dermatological care in patients with psoriasis.

Taken together, these papers provide a cohesive and multidimensional narrative supporting the reconceptualization of inflammatory skin diseases as systemic immunometabolic disorders with multi-organ implications (Figure 1). Advancing this integrated perspective will be essential for the development of precision medicine strategies capable of addressing not only cutaneous inflammation but also its long-term systemic consequences.

## Figures and Tables

**Figure 1:**
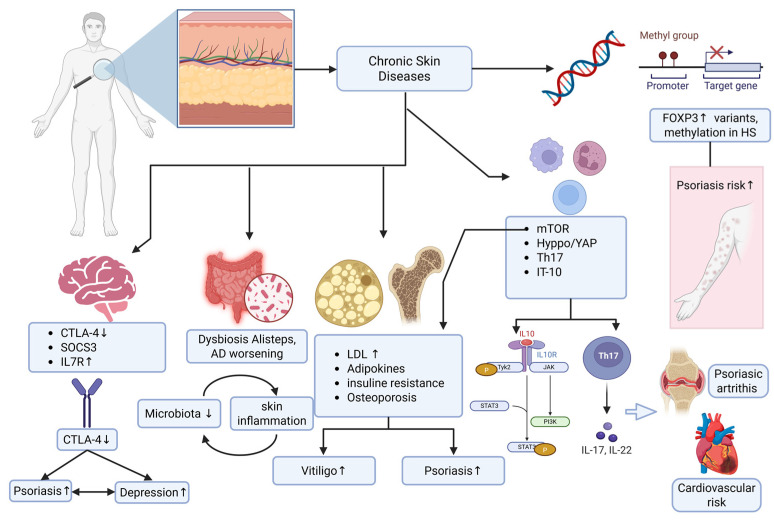
Schematic overview of the systemic molecular mechanisms implicated in chronic inflammatory skin diseases discussed in this Special Issue.
